# Continent Cutaneous Urinary Diversion With Augmentation Cystoplasty and Bladder Neck Closure for a Single-System Ectopic Ureter in an Adolescent Girl: A Report of a Rare Case

**DOI:** 10.7759/cureus.114796

**Published:** 2026-08-19

**Authors:** Preet Jain, Piyush P Singhania, Swapnil Nikam, Dhaval K Desai

**Affiliations:** 1 Urology, Mahatma Gandhi Mission Institute of Health Sciences, Navi Mumbai, IND; 2 Urology, Mahatma Gandhi Mission Institute of Health Sciences, Navi mumbai, IND

**Keywords:** augmentation cystoplasty, bladder neck closure, continent cutaneous urinary diversion, ectopic ureter, mitrofanoff procedure, pediatric reconstructive urology, pediatric urology in india, pediatric urology surgery, solitary functioning kidney, ureteric reimplantation

## Abstract

A single-system ectopic ureter draining a solitary functioning kidney is a rare cause of lifelong continuous urinary incontinence in girls and may be accompanied by a hypoplastic, poorly compliant bladder and a deficient bladder neck. This anatomy renders urethral continence unattainable and places the only kidney at risk, demanding complex lower urinary tract reconstruction.

A 16-year-old girl presented with continuous urinary incontinence since birth and inability to void a stream. Imaging showed an absent right kidney and a solitary, grossly hydronephrotic left kidney with cortical thinning, drained by a dilated, tortuous single ectopic ureter opening near the bladder neck. Cystoscopy revealed a low-capacity bladder (~40 mL) with an incompetent bladder neck. Serum creatinine (2.5 mg/dL) improved to 1.4 mg/dL after a left percutaneous nephrostomy. She underwent single-stage reconstruction comprising ileal augmentation cystoplasty, bladder neck closure, left ureteric reimplantation, and an appendico-caecal Mitrofanoff continent catheterisable channel brought out as a right-lower-quadrant V-flap stoma. The postoperative course was uneventful, and clean intermittent catheterisation via the stoma was established by the sixth postoperative week.

Continent cutaneous urinary diversion with augmentation cystoplasty provides a durable solution that restores continence, creates a low-pressure reservoir, and preserves renal function in a patient with a solitary kidney, achieving social continence and improved quality of life.

## Introduction

An ectopic ureter is one that drains at a site other than the bladder trigone. In girls, the orifice frequently lies distal to the sphincteric mechanism or within the genital tract, producing continuous urinary leakage superimposed on otherwise normal voiding. Most ectopic ureters arise from the upper moiety of a duplex system; a single-system ectopic ureter, in which the sole ureter draining a kidney is ectopic, is far less common and may be accompanied by maldevelopment of the trigone, bladder neck, and bladder, occasionally to the point of bladder agenesis [1]. In girls, this anomaly characteristically presents as lifelong continuous incontinence on a background of an otherwise normal voiding pattern, and because a normally sited urethral meatus is preserved, the diagnosis is frequently delayed into later childhood or adolescence [2]. The affected ureter may drain a solitary, dysplastic, or atrophic but still-functioning kidney, which further complicates management and heightens the imperative to preserve renal function [3].

This association is embryologically grounded. A ureteric bud arising too cranially on the mesonephric duct is delayed in its incorporation into the urogenital sinus, impairing the mesenchymal ingrowth required for normal trigonal and bladder neck development, so that the trigone may be deficient or absent and the bladder neck hypoplastic [4]. When such an ectopic ureter drains a solitary functioning kidney, the reconstructive problem is magnified, because simple reimplantation neither restores continence nor protects the only kidney from a small, high-pressure, poorly compliant reservoir.

Durable continence and renal preservation therefore depend on converting the bladder into a large-capacity, low-pressure reservoir and on providing a continent route for emptying. Augmentation cystoplasty using a detubularised ileal segment achieves the former, lowering storage pressure and protecting the upper tract [5]. Bladder emptying is then maintained by clean intermittent catheterisation, the technique that transformed the management of the non-emptying bladder [6]. When the native urethra cannot be made continent, the Mitrofanoff principle supplies a continent, easily catheterisable cutaneous channel that bypasses the urethra altogether [7]. We describe a single-system ectopic ureter draining a solitary functioning kidney in an adolescent girl with a hypoplastic bladder and an incompetent bladder neck, managed by single-stage continent cutaneous urinary diversion. This report follows the CARE guidelines for case reports [8].

## Case presentation

History and examination

A 16-year-old girl presented with continuous urinary incontinence since birth and an inability to produce a urinary stream. She had no other significant medical history, and her menstrual history was regular. Abdominal examination was unremarkable. Local examination revealed a normally sited urethral meatus with continuous dribbling of urine.

Investigations

Haemoglobin was 10.9 g/dL, and the total leucocyte count was 9,620/mm³. Serum urea was 50 mg/dL, and serum creatinine was 2.5 mg/dL. Urine routine microscopy showed 1-2 red blood cells and 1-2 pus cells per high-power field with occasional epithelial cells, and the urine culture was sterile.

Ultrasonography of the kidneys, ureters, and bladder demonstrated an absent right kidney and a solitary left kidney measuring 11.5×5.6 cm with gross hydronephrosis and cortical thinning (7 mm at the upper and mid poles and 8 mm at the lower pole); the bladder was empty.

**Figure 1:**
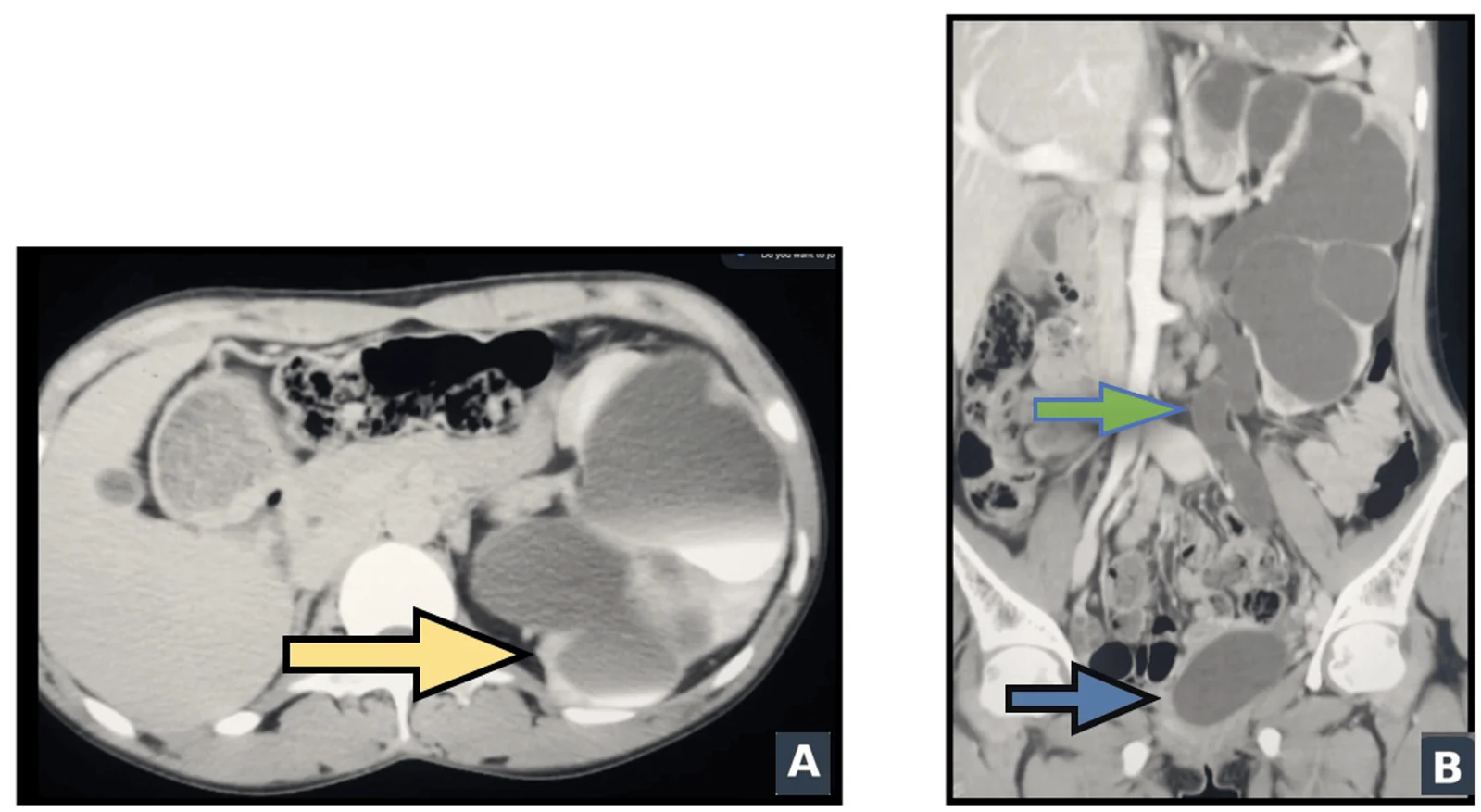
Preoperative computed tomography imaging: (A) axial section showing gross hydronephrosis with pooling of contrast on delayed phase (yellow arrow) and (B) coronal section showing dilated tortuous ureter (green arrow) and dilated lower ureter mimicking bladder (blue arrow) which was confirmed on retrograde pyelography

Computed tomography (CT) intravenous urography confirmed gross left hydronephrosis with significant parenchymal thinning and a dilated, tortuous left ureter opening into a bladder-like structure on the left lateral aspect, consistent with a single-system ectopic ureter (Figure 1).

**Figure 2:**
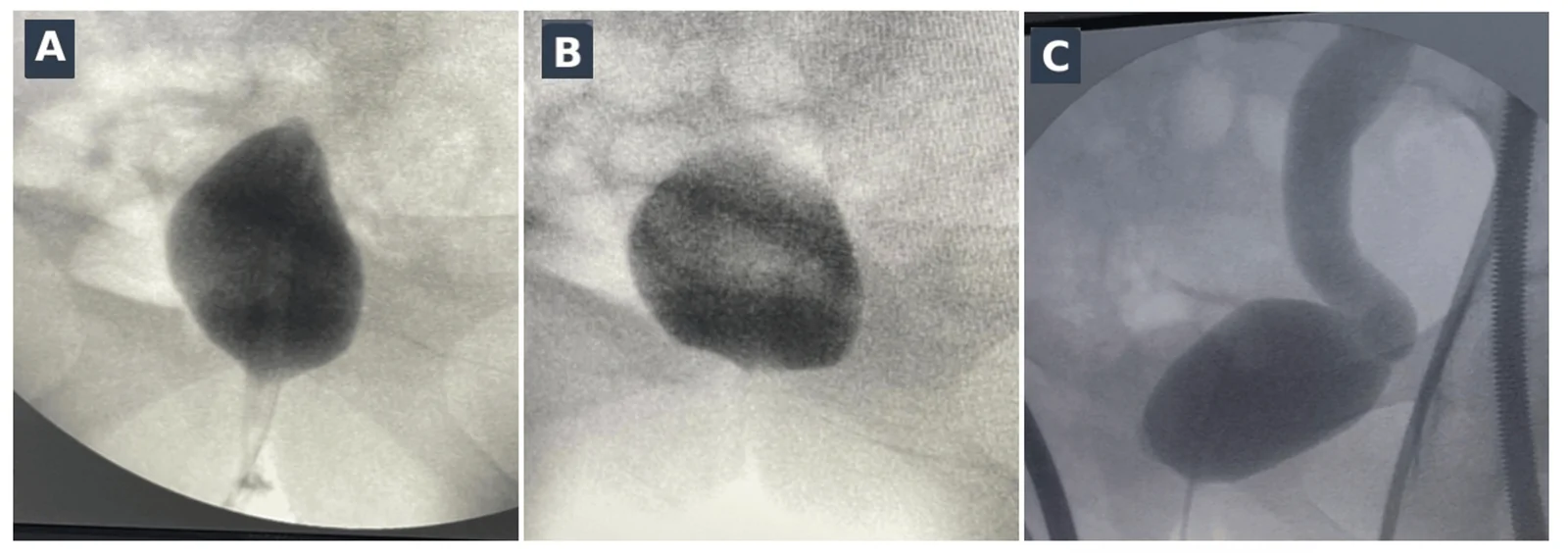
Intraoperative imaging. (A-B) Retrograde urethrography with micturating cystourethrography showing a low-volume bladder. (C) Retrograde pyelography demonstrating a left dilated, tortuous ectopic ureter

Diagnostic cystoscopy with retrograde pyelography and micturating cystourethrography (Figure 2A-2B) revealed a low-volume bladder (approximately 40 mL) with an incompetent bladder neck. The right ureteric orifice could not be visualised, and the left ureteric orifice lay at the edge of the meatus. Retrograde pyelography (Figure 2C) revealed a grossly dilated tortuous left ectopic ureter.

In view of the raised serum creatinine, a left percutaneous nephrostomy was performed, after which the creatinine fell to 1.4 mg/dL.

Diagnosis and surgical planning

The findings established a single-system ectopic ureter draining a solitary functioning (left) kidney, associated with a hypoplastic, poorly compliant bladder and a deficient bladder neck. Each anatomical problem was matched to a specific reconstructive solution (Table 1), and a single-stage continent cutaneous urinary diversion was planned.

**Table 1:** Clinical problems and corresponding reconstructive solutions

Clinical problem	Surgical solution
Small, poorly compliant bladder	Augmentation cystoplasty (ileal patch)
Undeveloped bladder neck and sphincter	Bladder neck closure
Left single-system ectopic ureter	Left ureteric reimplantation
Need for clean intermittent catheterisation	Mitrofanoff continent catheterisable channel

Operative procedure

**Figure 3:**
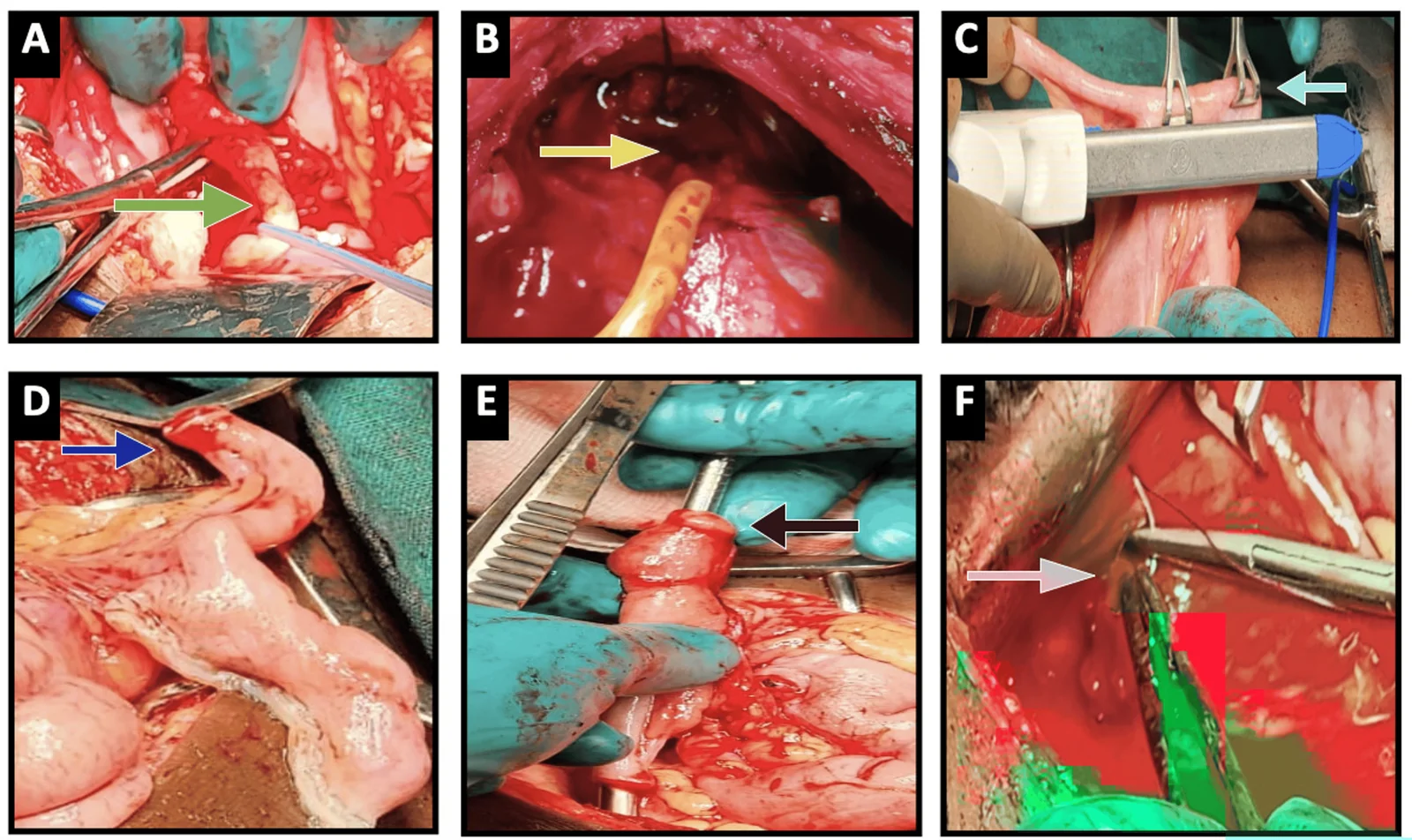
Key operative steps. (A) Solitary left ureter. (B) Bladder neck closure done. (C) Using a gastrointestinal anastomosis stapler, appendico-caecal tube was created. (D) Appendico-caecal catheterisable channel. (E) Dilated up to 12 Fr using Hegar's dilator. (F) Mitrofanoff channel spatulated and fixed to the bladder via submucosal tunnelling

Through a lower midline incision, a small, contracted bladder with a solitary left ureter was identified, and the ureter was divided most distally. The urethra was divided and closed at the bladder neck to establish continence. The appendix was opened at its distal end and, together with a segment of caecum, fashioned into a continent catheterisable channel; using a gastrointestinal anastomosis (GIA) stapler, the appendix with a 4-cm caecal cuff was divided while preserving its mesentery. The distal appendix was spatulated, passed through a submucosal tunnel into the bladder, and fixed. After dilatation with Hegar dilators, a 12-Fr Foley catheter passed freely through the appendico-caecal channel, Mitrofanoff implantation was done, and catheterisation was confirmed with 12-Fr catheterisation (Figure 3).

**Figure 4:**
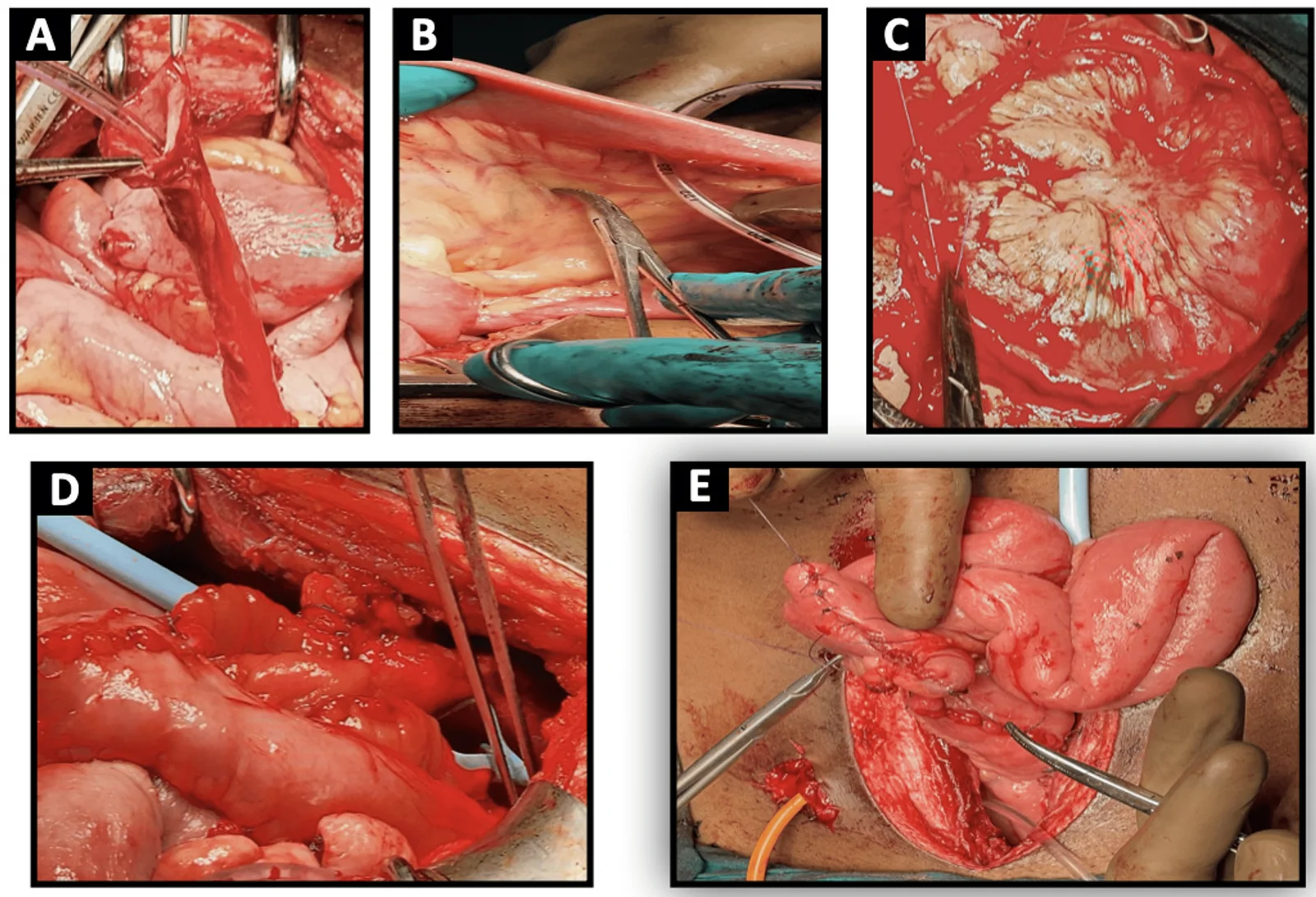
Key operative steps. (A) Left ureteric reimplantation. (B) 30 cm of ileum harvested 20 cm proximal to the ileocaecal junction. (C) Detubularised ileal segment in "U" configuration. (D) 18-Fr Malecot catheter placed. (E) Augmentation cystoplasty completed and Mitrofanoff channel brought out by creating a "V" flap stoma in the right lower quadrant of the abdomen with a Foley catheter in situ

Left ureteric reimplantation was performed into the native bladder over a 10-Fr infant feeding tube. A 30-cm ileal segment was harvested 20 cm proximal to the ileocaecal junction, opened along its antimesenteric border, folded in a "U" configuration, and sutured together with 4-0 polyglactin. This detubularised ileal patch was anastomosed to the bladder to complete the augmentation, and an 18-Fr Malecot catheter was placed and secured with 3-0 chromic catgut, creating an augmented low-pressure reservoir. The distal end of the continent channel was brought out as a "V" flap cutaneous stoma in the right lower quadrant with a 12-Fr catheter in the Mitrofanoff; a 10-Fr ureteric stent, the 18-Fr Malecot catheter, and a 24-Fr abdominal drain in the pelvic cavity completed the procedure (Figure 4).

Postoperative course

The postoperative recovery was uneventful and proceeded along a planned schedule of catheter and drain removal, culminating in the establishment of clean intermittent catheterisation through the continent stoma (Table 2). The stoma was sited at a cosmetically acceptable location.

**Table 2:** Postoperative timeline of catheter and drain management POD: postoperative day

Day	Intervention
POD 4	Clamped percutaneous nephrostomy removed
POD 6	Ureteric stent (infant feeding tube) removed
POD 8	Abdominal drain removed
POD 15	Bedside stomal mucosal debridement under local anaesthesia
POD 42	Mitrofanoff catheter removed and clean intermittent catheterisation started
POD 46	Malecot catheter removed after a three-day Foley trial via the Mitrofanoff channel

Follow-up and outcome

At discharge, the patient was advised clean intermittent self-catheterisation every four hours through the stoma using a Tiemann catheter, with a once-daily bladder wash through the same catheter. She was counselled to maintain a high oral fluid intake and a salt-rich, high-protein diet and was prescribed oral sodium bicarbonate to offset the metabolic acidosis associated with bowel segment reservoirs.

Renal function was scheduled for three-monthly follow-up with ultrasonography and serum creatinine along with venous blood gas monitoring six-weekly for three months and then six-monthly thereafter. Her follow-up serum creatinine was 1.4 mg/dL. The reconstruction achieved reliable continence via the stoma with a comfortable dry interval, while the augmented low-pressure reservoir and the improvement in serum creatinine after drainage reflected the preservation of the solitary kidney.

The patient underwent surgery in October 2025 and was followed up after 1.5 months (December 2025) with venous blood gas and renal function tests, which suggested normal venous blood gas findings and a serum creatinine of 1.4 mg/dL. After three months (January 2026), the patient underwent ultrasonography and venous blood gas and renal function tests. The ultrasonography was suggestive of a left solitary kidney with moderate hydronephrosis (decreased), parenchymal thinning (similar), and the rest of the findings being almost consistent with the previous findings.

## Discussion

A single-system ectopic ureter draining a solitary functioning kidney is an uncommon anomaly, and its coexistence with a hypoplastic bladder and a deficient bladder neck is rarer still [1]. The continuous incontinence that brings these patients to attention reflects an orifice lying beyond the sphincteric mechanism, while preserved sensation and a normally sited meatus can delay recognition for years, as in our patient [2]. The same embryological error that displaces the ureteric orifice also limits trigonal and bladder neck development, so the bladder is typically small, poorly compliant, and incapable of continence on its own.

These features explain why an isolated procedure is often insufficient. Reimplanting the ectopic ureter restores drainage but leaves the incompetent outlet and the high-pressure, low-capacity reservoir unaddressed, either of which would jeopardise a solitary kidney. The primacy of the reservoir is underscored by Stavrinides et al., who, in children with single-system ectopic ureters, found that continence after reimplantation correlated with preoperative bladder capacity, with the smallest bladders failing to achieve continence by reimplantation alone [9]. Where the bladder attains adequate capacity, timely ureteric reimplantation on its own can restore both continence and bladder function without diversion [4]; in our patient, however, the tiny (~40 mL) hypoplastic bladder together with an absent functional bladder neck made this route untenable and warranted definitive continent reconstruction, as others have advocated when the native outlet is unsalvageable [10]. Our strategy therefore combined four complementary components. Augmentation cystoplasty with a detubularised ileal patch converts the bladder into a large-capacity, low-pressure reservoir that protects the upper tract [5]. Bladder neck closure secures continence where the native sphincter cannot be salvaged. Ureteric reimplantation re-establishes non-refluxing drainage of the solitary kidney. Finally, a continent catheterisable channel provides a route for emptying that is maintained by lifelong clean intermittent catheterisation [6].

The Mitrofanoff principle, first described in 1980, established that a low-pressure reservoir emptied through a continent, catheterisable stoma could replace urethral voiding [7]. Bladder neck closure combined with enterocystoplasty and a Mitrofanoff channel has since been shown to achieve continence reliably in complex cases, as either primary or salvage therapy, albeit at the cost of committed lifelong catheterisation and a measurable rate of surgical re-intervention [11]. Duckett and Snyder broadened the concept, pairing a compliant reservoir with antireflux ureteric implantation and a range of catheterisable conduits, and it has since become a cornerstone of lower tract reconstruction [12]. Large series with long-term follow-up confirm that the appendix is a durable continence channel, with stomal stenosis the most frequent late complication requiring vigilance [13]. Long-term follow-up of Mitrofanoff conduits similarly demonstrates durable continence, with most complications relating to the stoma and tending to decline after the first decade [14]. When the appendix is absent or unsuitable, a transversely retubularised ileal (Yang-Monti) segment offers an effective alternative efferent conduit [15]. In our patient, an appendico-caecal channel was used and functioned well, with clean intermittent catheterisation established by the sixth postoperative week.

Because the result depends on lifelong catheterisation and on surveillance for the metabolic and reservoir-related consequences of incorporating bowel into the urinary tract, careful patient selection, education, and structured follow-up are essential. The principal limitation of this report is its single-case nature and short follow-up; nonetheless, it demonstrates that, even with a solitary functioning kidney, a complex congenital lower urinary tract anomaly can be reconstructed in a single stage to restore continence and preserve renal function.

## Conclusions

Continent cutaneous urinary diversion with augmentation cystoplasty, bladder neck closure, ureteric reimplantation, and a Mitrofanoff channel offers a durable, single-stage solution for a single-system ectopic ureter associated with a hypoplastic bladder and an incompetent bladder neck in a patient with a solitary functioning kidney. It provides reliable continence through the stoma, improves bladder compliance and capacity by creating a low-pressure reservoir, preserves renal function, and restores a socially normal life through a dependable dry interval and improved quality of life.
